# Epigenetic Suppression of the IL-7 Pathway in Progressive Glioblastoma

**DOI:** 10.3390/biomedicines10092174

**Published:** 2022-09-02

**Authors:** Marton Tompa, Zoltan Kraboth, Bence Galik, Bela Kajtar, Attila Gyenesei, Bernadette Kalman

**Affiliations:** 1 Szentagothai Research Center, University of Pecs, 7624 Pecs, Hungary; 2 Department of Laboratory Medicine, University of Pecs, School of Medicine, Clinical Center, 7624 Pecs, Hungary; 3 Department of Pathology, University of Pecs, School of Medicine, Clinical Center, 7624 Pecs, Hungary; 4 Department of Clinical Molecular Biology, Medical University of Bialystok, 15-269 Bialystok, Poland

**Keywords:** glioblastoma, immune evasion, epigenomics, CpG methylation, interleukin 7 pathway

## Abstract

Background: Immune evasion in glioblastoma (GBM) shields cancer cells from cytotoxic immune response. Methods: We investigated CpG methylation in promoters, genes, and pathways in 22 pairs of formalin-fixed paraffin-embedded sequential (FFPE) GBM using restricted resolution bisulfite sequencing (RRBS) and bioinformatic analyses. Results: Gene ontology revealed hypermethylation in elements of the innate and adaptive immune system when recurrent GBM samples (GBM^rec^) were compared to control (CG) and primary GBM samples (GBM^prim^). Higher methylation levels of the IL-7 signaling pathway and response to IL-7 were found in GBM^rec^ suggesting a progressive blockade of the IL-7 driven T cell response in sequential GBM. Analyses of the Cancer Genome Atlas array-based data confirmed hypermethylation of the IL-7 pathway in recurrent compared with primary GBM. We also quantified DNA CpG methylation in promoter and gene regions of the IL-7 ligand and IL-7 α-receptor subunit in individual samples of a large RRBS-based sequential cohort of GBM in a Viennese database and found significantly higher methylation levels in the IL-7 receptor α-subunit in GBM^rec^ compared with GBM^prim^. Conclusions: This study revealed the progressive suppression of the IL-7 receptor-mediated pathway as a means of immune evasion by GBM and thereby highlighted it as a new treatment target.

## 1. Introduction

Glioblastoma (GBM) is one of the most severe and lethal primary brain tumors. During its development, tumor cells accrue somatic genomic rearrangements, mutations, and copy number alterations that are accompanied by changes in epigenomic and gene expression profiles [1,2]. The first comprehensive epigenomic analysis used array-based CpG methylation data to show segregation of GBM into molecular subgroups in overlap with the ones revealed by integrated genomic and transcriptomic analyses [2,3]. Subsequently, several targeted or comprehensive epigenomic studies showed differential CpG methylation in genes of gliomas of various grades and subtypes compared with each other or to non-tumorous brain tissues. Of particular interest, epigenomic effects of the oncometabolite 2-OH-glutarate were revealed in isocitrate dehydrogenase (IDH) mutant compared with IDH wild-type gliomas [3,4]. Relatively fewer studies used sequencing for studying differential methylation in gliomas, particularly in formalin-fixed, paraffin-embedded (FFPE) tissues that are predominantly available in the clinical setting. In addition, due to the aggressive nature of GBM, information regarding the epigenomic profiles in primary and recurrent GBM samples is somewhat scarce. In a previous study, relying on the Restricted Resolution Bisulfite Sequencing (RRBS) technology, we detected several differentially methylated pathways (DMP) in primary and recurrent FFPE GBM specimens [5]. Subsequently, we further investigated the most outstanding DMPs, the Wnt and catecholamine pathways, by comparing the promoter and gene methylation profiles with protein expression levels of their elements in the same specimens [6,7]. In the present study, we investigated in more depth the third-most outstanding DMP, the immune pathways that have not been previously scrutinized [5]. The reason why we opted to highlight the epigenomic regulation of immune pathways in progressive GBM pairs is related to several considerations. Mechanisms of immune evasion have been well understood in various tumors, including GBM [8]. However, while immune checkpoint inhibitors or other means of immune therapies have been quite successful in many cancers [9,10], such approaches have largely failed and the reason for that is only partially understood in GBM [11]. Molecular engineering, however, may improve treatment efficacy by incorporating and combining well-selected immune pathway elements that are suppressed by the tumor. If we can pinpoint some of those elements that are not only suppressed by the tumor (i.e., by epigenomic regulation) but may also be beneficially manipulated by novel treatment strategies, we can strengthen the development of future immune therapies in GBM.

## 2. Materials and Methods

### 2.1. Subjects of the Study

After routine histopathological evaluations between 1999 and 2017, the leftover FFPE GBM samples were archived at the Department of Pathology, University of Pecs. None of the patients were alive at the time of the initiation of the original epigenomic analyses in 2018–2019 [5]; the histological diagnoses of the obtained tumor specimens were based on the 2016 WHO guideline [12]. As additional immunohistochemical studies were subsequently also performed and reported on the same tissue specimens [5,6,7], no sufficient tissue remained for complementary testing of those molecular alterations (i.e., H3F3A, HIST1H3B, HIST1H3C, TERT, EGFR-amplification) recommended in the 2021 WHO revision [13]. As Table S1 shows, however, all tumors were late-onset GBMs, and thus mutations in histone proteins would be very unlikely. The Regional Clinical Research Committee of the University of Pecs approved the study (Number: 7517 PTE 2018 and 2019) in compliance with the Code of Ethics of the World Medical Association (Declaration of Helsinki).

### 2.2. GBM Cohort

The epigenomic cohort included 22 primary (GBM^prim^) and recurrent (GBM^rec^) pairs of IDH-1 R132H mutation-negative GBMs without G-CIMP (glioma CpG island methylator phenotype) [5]. The rarity of other IDH-1 and IDH-2 mutations and the absence of G-CIMP make it unlikely that IDH-mutant GBM samples were included in our cohort. GBM^prim^ samples were obtained from the first surgery, before chemo- and irradiation therapy, while GBM^rec^ samples were surgically obtained at recurrence, after chemo- and irradiation therapy. Of the 14 male and 8 female patients, documentation could be accessed for 18 patients receiving temozolomide-based chemo- and irradiation therapy after the first surgery, while no therapy-related information could be gathered in 4 cases. Table S1 summarizes the patients’ characteristics.

### 2.3. Control Group

Methylomes of five epilepsy surgery specimens (CG) obtained from the European Nucleotide Archive [14] (https://www.ebi.ac.uk/ena, Primary Accession: PRJNA391429; EGAS00001002538 accessed on 17 June 2022) were used as controls in the DNA CpG methylation analyses in comparisons with GBM^prim^ and GBM^rec^ [5,6,7].

### 2.4. DNA Isolation and Bisulfite Sequencing

Details of DNA isolation, library preparation, CpG methylation profiling, and bioinformatics analyses have been previously described [5]. In brief, DNA was extracted using the QIAamp DNA FFPE Tissue Kit (Qiagen GbmH, Hilden, Germany) from sections of FFPE blocks. Bisulfite-converted libraries were prepared using the Premium Reduced Representation Bisulfite Sequencing kit (Premium RRBS Kit 24x, Diagenode SA, Seraing, Belgium) and sequenced applying the Illumina NextSeq 500/550 High Output Kit v2.5 (75 cycles) on a NextSeq 550 machine (Illumina, San Diego, CA, USA) [5].

### 2.5. Validation Cohort-1

The TCGA validation cohort included array-based CpG methylation results from 12 pairs of fresh-frozen, IDH wild-type primary and recurrent GBMs tested on the Illumina Human Methylation 450 arrays [15] (https://portal.gdc.cancer.gov/repository?filters=%7B%22op%22%3A%22and%22%2C%22content%22%3A%5B%7B%22op%22%3A%22in%22%2C%22content%22%3A%7B%22field%22%3A%22cases.project.project_id%22%2C%22value%22%3A%5B%22TCGA-GBM%22%5D%7D%7D%2C%7B%22op%22%3A%22in%22%2C%22content%22%3A%7B%22field%22%3A%22files.data_category%22%2C%22value%22%3A%5B%22DNA%20Methylation%22%5D%7D%7D%5D%7D&searchTableTab=cases accessed on 18 December 2019).

### 2.6. Validation Cohort-2

The second validation cohort included RRBS sequence data from 112 pairs of FFPE primary and recurrent GBM tumors with IDH-1 wild-type status (https://www.ebi.ac.uk/ena, Primary Accession: EGAS00001002538, Secondary Accession: PRJNA391429) [14].

### 2.7. Bioinformatics and Statistics

Bioinformatics analyses of the cohorts are detailed elsewhere [5]. First, a quality control step by FastQC was carried out, followed by the removal of low-quality bases and adapters using TrimGalore. The Bismarck program was used to map bisulfite-converted sequences to the GRCh37 reference genome and to perform methylation calls. The differentially methylated CpG sites, genes, and pathways were defined by RnBeads. The rank-based GO (gene ontology) enrichment analysis was conducted in various rankings (e.g., best 100, 500, 1000 and automatic cutoff) in order to compensate for possible sample quality issues.

An in-house-generated R script allowed us to extract and compare DNA CpG methylation data from the Bismark analysis results within predefined genomic regions, and to plot the data in the selected regions in individual samples or sample sets. When methylation levels of our selected markers were quantified in individual samples (complementing the cohort level analyses), we chose to assess CpG methylation within the promoter + gene regions instead of only within the promoter regions. The reason for that was related to the fragmentation of DNA in the FFPE specimens. We observed that analyzing CpG sites only within the (approx. 2000 bp) gene promoters in our relatively small cohorts could not provide statistically meaningful outcomes for defining differential methylation [6,7]. The methylation levels were expressed as percentages where the numerators were calculated from the numbers of methylated sites (with the degree of methylation also computed into the figures), and the denominators were the numbers of all CpG sites within the investigated region, and multiplied by 100.

Differences in methylation levels within promoter + gene regions in pairs of GBM^prim^ and GBM^rec^ were compared using the Wilcoxon signed rank test, while of those in the CG and GBM^prim^ or GBM^rec^ samples were tested using the Mann–Whitney U test in the SPSS v. 23.0 package (SAGE, IBM^®^ SPSS^®^ Statistics v23.0).

## 3. Results

### 3.1. Differential DNA CpG Methylation of Immune Pathways in Paired GBM and Control Specimens (Table 1, Tables S2 and S3)

GO analyses of the bisulfite converted DNA sequences in CG and IDH-wild-type GBM specimens at initial diagnosis (GBM^prim^) or at recurrence (GBM^rec^) revealed several differentially methylated pathways [5], including various immune pathways, the focus of the present analyses. We feature here DMP immune elements that have not been previously reported (Table 1).

**Table 1:** Differential methylation in innate and adaptive immune pathways, and in the IL-7 initiated pathway in our cohort: this table presents summary data extracted from the RnBeads GO analyses shown in details in Table S2. For easier review, we here combined the various immune pathway data into three groups: innate and adaptive immune pathways, and separately show data for the IL-7 initiated pathway. While both hypo- and hypermethylation are seen in the GBM^prim^ vs. CG, GBM^rec^ vs. CG and GBM^rec^ vs. GBM^prim^ comparisons of the three immune pathways, suggesting intratumor heterogeneity, it is noteworthy that the shift towards hypermethylation is much more significant during tumor progression.

More Methylated Promoter and/or Gene within Pathways in the First Cohort Group Compared to the Second (Reference) Cohort Group	*p*-Value Range	Less Methylated Promoter and/or Gene within Pathways in the First Cohort Group Compared to the Second (Reference) Cohort Group	*p*-Value Range
GBM^prim^ vs. CG		GBM^prim^ vs. CG	
Innate immune response (regulation of innate immune response, positive regulation of interleukin-6-mediated signaling pathway, positive regulation of macrophage cytokine production)	0.006–0.0084	Innate immune response (immune system development, regulation of cytokine-mediated signaling pathway)	0.0032–0.0073
Adaptive immune response (regulation of acute inflammatory response to antigenic stimulus)	0.0084	Adaptive immune response (positive regulation of CD8-positive-alpha-beta cytotoxic T cell extravasation, B and T cell receptor signaling pathway, T cell homeostasis, negative regulation of B cell activation, regulation of cytokine-mediated signaling)	0.0019–0.0073
Interleukin-7-mediated signaling pathway and response to interleukin-7	0.0001	Interleukin-7-mediated signaling pathway and response to interleukin-7	0.0027
GBM^rec^ vs. CG		GBM^rec^ vs. CG	
Innate immune response (interleukin-6 mediated signaling pathway and response to interleukin 6, interleukin-11-mediated signaling pathway, positive regulation of NK T cell activation)	0.0003–0.0092	Innate immune response (positive regulation of myeloid leukocyte mediated immunity)	0.0023
Adaptive immune response (Interleukin-27-mediated signaling pathway, T cell differentiation in the thymus, Interleukin-11-mediated signaling pathway, positive regulation of T-helper 2 cell cytokine production)	0.0009–0.0059	Adaptive immune response (negative regulation of immature T-cell proliferation in thymus, positive regultation of immunoglobulin mediated immune response, immune response to tumor cells)	0.0051–0.0082
Interleukin-7-mediated signaling pathway and response to interleukin-7	0	Interleukin-7-mediated signaling pathway and response to interleukin-7	0.0007
GBM^rec^ vs. GBM^prim^		GBM^rec^ vs. GBM^prim^	
Innate immune response (protection from natural killer cell mediated cytotoxicity, negative regulation of innate immune response and cytokine production)	0.0069–0.0097	Innate immune response (negative regulation of cytokine secretion, susceptibility to and positive regulation of natural killer cell mediated cytotoxicity, regulation of cell killing, positive regulation of leukocyte mediated immunity and cytotoxicity, negative regulation of interleukin-1 alpha production)	0.0006–0.0056
Adaptive immune response (antigen processing and presentation of endogeneous peptides, CD4-positive or CD8-positive alpha-beta T cell lineage commitment and proliferation, positive regulation of T cell mediated immunity, negative regulation of alpha-beta T cell proliferation)	0.0011–0.0098	Adaptive immune response (regulation of dendritic cell antigen processing and presentation, T cell homeostasis, regulation of lymphocyte mediated immunity, T cell mediated cytotoxicity, positive regulation of humoral immune response mediated by circulating immunoglobulin)	0.0002–0.0093
Interleukin-7-mediated signaling pathway and response to interleukin-7	0	Interleukin-7-mediated signaling pathway and response to interleukin-7	0.0023

First, looking into differentially methylated pathways based on promoters or genes at all (100, 500, and 1000) ranks, we detected more methylated immune pathways including antigen processing and presentation of endogenous peptides, positive regulation of CD4+ and CD8+ alpha-beta T cell proliferation, and negative regulation of NK cell activation in GBM^rec^ compared with GBM^prim^. This observation suggests a higher activity of the adaptive immune response in GBM^prim^ than in GBM^rec^, possibly related to its progressive evasion by the tumor or to the immune suppressive effects of chemo- and radiotherapy (Table S2). However, making the same sequential sample comparisons at the automatic cut off rank, hypermethylation of the interleukin-7 (IL-7) signaling pathway and response to IL-7 are also indicated in GBM^rec^, suggesting that suppression (higher methylation) of the IL-7-driven T cell maturation and response may be part of the tumor’s progressive immune evasion or the result of a therapeutic immune suppression. This notion is further supported by the observation that the IL-7-mediated signaling pathway based on methylation in either the promoters or genes is significantly suppressed in GBM^rec^ compared with CG (Table 1 and Table S2).

The analysis of the TCGA array-based methylation data also revealed that the IL-7 signaling pathway and responses to IL-7 are significantly more methylated in the recurrent than in the primary samples of GBM. This consensus in the two data sets is quite remarkable given the small numbers of sequential GBMs in our cohort (22 pairs) as well as in the TCGA cohort (12 pairs) (Table S3).

The analyses of hypomethylated pathways also revealed the involvement of innate and adaptive immune system when GBM^prim^ vs. CG, GBM^rec^ vs. CG, or GBM^rec^ vs. GBM^prim^ were compared (Table 1, Tables S2 and S3). Based on the analyses of promoters, lower levels of methylation in the IL-7-mediated signaling pathway and response to IL-7 were noted in the GBM^rec^ vs. GBM^prim^, and in the GBM^prim^ vs. CG or GBM^rec^ vs. CG comparisons, but with much lower levels of significance than in the hypermethylated pathway comparisons. This latter finding is likely related to the well-known clonal and biological intratumor heterogeneity of GBM samples. Curiously, the observations regarding the hypomethylated pathways were also partly supported by the outcome of TCGA array-based data (Table 1, Tables S2 and S3).

### 3.2. Differential Methylation of CpGs in Individual Promoter + Gene Regions of IL-7 and IL-7 Receptor in GBM Cohorts

The differential methylation in the IL-7 pathway in primary and recurrent GBM pairs of our own cohort and the TCGA cohort in the GO analyses prompted us to assess the DNA CpG methylation levels in the promoter + gene regions of IL-7 and its receptor (α subunit) in individual samples over time.

However, this analysis could not reveal differential methylation levels in the aforementioned regions when GBM^rec^ and GBM^prim^ samples were compared to each other or to the CG samples due to the small size of our FFPE GBM cohort (22 pairs of specimens).

Similarly, the array data from the 12 pairs of TCGA GBM cohort did not provide sufficient information of CpGs in the investigated IL-7 and IL-7Rα promoter + gene regions to allow us to obtain reliable statistical outcomes when assessing differential methylation levels in these regions of individual sample pairs.

Therefore, we looked into the RRBS-based sequence data of 112 primary and recurrent GBM pairs from the study by Klughammer et al. [16] and statistically tested the differential CpG methylation in the IL-7 and IL-7Rα promoter + gene regions in individual samples (https://www.ebi.ac.uk/ena, Primary Accession: PRJNA391429; EGAS00001002538, accessed on 17 June 2022) [14]. Comparing the 112 GBM^rec^ and GBM^prim^, we found a significantly higher methylation level in the IL-7 α-receptor subunit (*p* = 0.041) in the GBM^rec^ compared with GBM^prim^ samples in consensus with the above-described GO results. However, no statistical differences in the methylation levels of IL-7 promoter + gene regions were detected in the same comparison, suggesting that the differential methylation in the IL-7 -mediated pathway and in the response to IL-7 detected by GO analyses are likely related to the differential methylation in the receptor (and possibly its downstream elements), but not in the ligand.

## 4. Discussion

In our previous studies, we used the RRBS technology to identify DMPs, and performed simultaneous epigenomic and protein expression analyses for the two most affected (Wnt and catecholamine) pathways in progressive GBM samples [5,6,7]. In this study, we presented the third group of the most differentially methylated pathways, the immune pathways in longitudinal GBM samples, to highlight a previously not reported DMP, the IL-7/IL-7 receptor pathway, with significant practical relevance.

In contrast to other solid tumors, treating GBM with immunotherapies is more challenging because of the blood–brain barrier, the high degree of intra- and inter-tumor heterogeneity, and the complexity of the tumor’s microenvironment (TME) that significantly contributes to immune evasion. Mechanisms of immune evasion in GBM include the tumor’s and TME’s expression of inhibitor proteins targeting CD4^+^ and CD8^+^ T cells, B and NK cells, the recruitment of immunosuppressive regulatory T cells and myeloid-derived suppressor cells, as well as the production of immunosuppressive cytokines that induce rapid T cell exhaustion and reduced T cell survival [8,10,11,17]. Epigenetic regulation in the tumor and the TME, but also in the infiltrative immune cells during the initial and progressive stages of gliomagenesis contribute to mechanisms of immune evasion. It is worth adding that infiltration by immune cells in GBM is typically moderate or scarce, as it was in the majority of our samples [5]. Several research groups have sought to develop an effective immune strategy to circumvent the GBM cells’ immune escape mechanisms. After the disappointing outcome of early growth factor receptor inhibition strategies, the newer approaches include immune checkpoint inhibitors, modulation of tumor infiltrating lymphocytes (TILs), and most recently, the engineered chimeric antigen receptors (CAR) carried by thymocytes (T) and natural killer (NK) cells [10,11]. Significant efforts have been made to equip tumor-infiltrating CAR-T cells with various interleukins to prolong their persistence and to enhance therapeutic efficacy against solid tumors [16]. The optimal T cell activation requires 3 main signals: 1: T- receptor activator; 2: co-stimulation; and 3: immune stimulatory cytokines, which are also considered in some forms of complementation in CAR T cell development [18,19].

**Figure 1:**
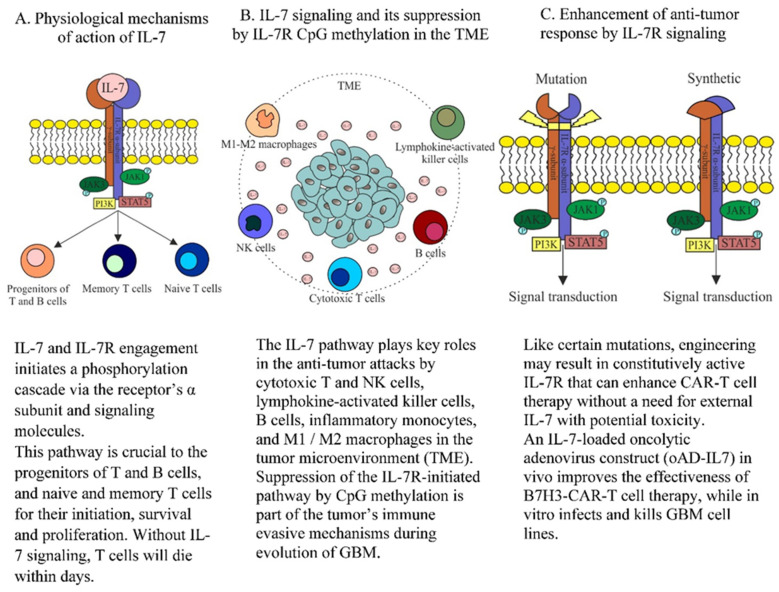
Physiological mechanisms, suppression, and potential therapeutic targeting of the IL-7-initiated pathway in GBM: the figure details (**A**) the normal mechanisms of action [22], (**B**) cell groups affected by epigenetic suppression, and (**C**) therapeutic utility of the IL-7R pathway in GBM [18,19,23].

Our GO analyses revealed significantly more methylation in pathways of the innate and adoptive immune system including different types of interleukins (e.g., 3, 7, 15, and 23) in the GBM^rec^ compared with GBM^prim^ samples. While epigenetic regulation contributing to immune evasion is a well described mechanism in GBM [20], increasing methylation of CpGs of relevant genes in progressive tumor samples has not been previously highlighted according to the literature. More importantly, higher methylation in the IL-7 signaling pathway and response to IL-7 were among the most prominent and novel observations when we compared GBM^rec^ samples to GBM^prim^, and GBM^rec^ samples to CG both in our own and in the TCGA validation cohorts. These previously unreported observations suggest a progressively increasing methylation, and thus, likely a gradual expression suppression in the IL-7 pathway when comparing CG, GBM^prim^, and GBM^rec^ (Table 1, Figure 1). IL-7 is a cytokine primarily produced by stromal and dendritic cells, but also by some neurons and glial cells in the central nervous system (Figure 1) [21].

The IL-7 receptor is a heterodimer consisting of an α and a common gamma chain subunit [24]. The engagement of the receptor pathway contributes to T and B cell development and to the survival of tumor-infiltrating T cells [21,25,26]. The DMP of IL-7 signaling and response in the primary and recurrent GBM pairs and in the CG group prompted us to evaluate the DNA CpG methylation levels in the promoter + gene regions of IL-7 and its receptor (α subunit) in individual samples. (We omitted the analysis of the γ receptor subunit since it is encoded on the X chromosome). While in our small cohort there were 22 pairs of FFPE samples and in 12 pairs the even smaller cohort of fresh-frozen TCGA samples, we had no power to test differential methylation of CpGs in the investigated regions in individual samples; we had access to the RRBS results of a larger cohort of 112 GBM^prim^ and GBM^rec^ by Klughammer et al. [16], where we detected a significantly higher methylation level in the IL-7 α-receptor subunit, but not in the IL-7 promoter + gene regions in the GBM^rec^ compared to GBM^prim^ samples. Altogether, these results underscore the increasing methylation in the IL-7 -mediated pathway and response to IL-7 involving the IL-7 receptor (and possibly its down-stream signaling elements) in progressive GBM (Figure 1). Determining whether the increasing methylation and likely expression suppression of the ILR-7 α- subunit is part of the tumor’s intrinsic mechanisms to avoid immune attacks, or the effect of chemo- and irradiation therapy, requires further in vitro analyses. Nevertheless, the progressive methylation of this key immune pathway may certainly be an important element of T cell inhibition, a finding previously not established by molecular analyses of GBM specimens.

Patients with GBM are generally treated according to the Stupp protocol often associated with significant hematologic toxicity and the development of TMZ resistance over time. Although molecular engineering may succeed to overcome these obstacles, these strategies are still in experimental stages [27]. Clinical immune therapies have mostly followed strategies successful in solid tumors outside of the brain, and largely have failed in GBM. Therefore, highlighting a key pathway in immune response that is epigenetically suppressed in ex vivo human GBM samples may serve more efficient development of biological therapies in the future. IL-7 has been identified as a key cytokine in the compensatory reaction to declining lymphocytes [28,29]. While exogenous administration of IL-7 could elevate the CD4+ and CD8+ T-cell counts, higher doses of this cytokine are associated with significant toxicity [30]. Therefore, manipulating the IL-7 receptor and its down-stream pathway may be more rewarding.

The importance of IL-7 signaling for sustaining CAR-T cell activity against solid tumors has been recognized, and led to the engineering of a constitutively active IL-7 receptor to enhance the adoptive CAR-T cell immunotherapy [31]. The applications of the C7R construct to GD2-CAR T cells and EphA2-CAR T cells effectively targeting metastatic neuroblastoma and orthotopic GBM in animal models, respectively, are particularly attractive since these approaches do not necessitate an external administration of IL-7 with potential debilitating cytotoxicity [18,19].

In another strategy, IL-7-loaded oncolytic adenoviruses (oAD-IL7) were used in vivo to improve the effectiveness of B7H3-CAR-T cell therapy by enhancing T cell persistence, which led to prolonged survival of the GBM tumor-bearing mice. This virus construct (oAD-IL7) in vitro also successfully infected all GBM cell lines, and induced apoptosis of tumor cells [23].

While our finding primarily suggests development of immune strategies enhancing the IL-7 receptor mediated pathway to diminish immune evasion in GBM, early results from other cancer treatment paradigms offer further possibilities. Selective epigenetic manipulation of target genes such as oncogenes and tumor suppressor genes has been promising in several tumors [32,33]. Epigenetic changes altering expression of interleukins or their receptors are known to be associated with diseases, which may be therapeutically modulated by targeting histone modification, DNA CpG methylation, or microRNA and long non-coding RNA molecules [34]. To our knowledge, similar strategies have not been reported, but may also be plausible to consider regarding the IL-7/IL-7 receptor pathway in GBM. As numerous mutations and epigenetic alterations collaborating with the IL-7 receptor signaling pathway have been identified in acute lymphoblastic leukemia, the IL-7 receptor interactome may also be of interest for advancing new treatment development in GBM [35]. Monitoring effectiveness of newer therapeutic interventions will also be of key importance, and may involve the inclusion of well-characterized biomarkers for liquid biopsy in addition to more traditional clinical and paraclinical measures [36].

Since our study involved FFPE human GBM samples, and the complementary analyses of array-based or sequence-based methylome data of databases were also derived from frozen or FFPE human gliomas, functional experiments with target manipulation remain to be conducted in the future. Nevertheless, the finding presented here is novel and strengthen concepts for new treatment development.

In conclusion, our study emphasizes the progressive suppression of the IL-7 pathway by CpG methylation in sequential GBM, suggesting that this mechanism may play a key role in immune evasion and supports its targeting by the engineering of novel immune therapies (Figure 1).

## Data Availability

Raw sequencing data were uploaded to the European Nucleotide Archive (https://www.ebi.ac.uk/ena, Primary Accession: PRJEB38380, Secondary Accession: ERP121800 accessed on 19 May 2020). Some of the data are also provided in the Electronic Supplementary Material. Publicly available bioinformatics programs and tools were used as detailed in the Manuscript. The in-house script used in this study is available at https://github.com/galikbence/MethylPatternViz (accessed on 1 November 2020).

## Supplementary Materials

The following supporting information can be downloaded at: https://www.mdpi.com/article/10.3390/biomedicines10092174/s1, Table S1. Patients’ characteristics: This table summarizes gender, age distribution (mean and median with interquartile rage values) and treatments of patients included in the study. Table S2. GO data from RnBeads of our own cohort of GBMprim, GBMrec and CG: This table captures differential methylation in immune pathways by GO analyses selecting either gene or promoter data and comparing GBMprim to CG, GBMrec to CG, and GBMrec to GBMprim. Table S3. GO data comparing primary and recurrent GBM in the TCGA database: This table presents differential methylation in immune pathways by GO analyses selecting either gene or promoter data and comparing recurrent to primary GBM in the TCGA database.

## References

[1] The Cancer Genome Atlas Research Network (2008). Comprehensive genomic characterization defines human glioblastoma genes and core pathways. Nature.

[2] Verhaak R.G., Hoadley K.A., Purdom E., Wang V., Qi Y., Wilkerson M.D., Miller C.R., Ding L., Golub T., Jill P. (2010). Integrated genomic analysis identifies clinically relevant subtypes of glioblastoma characterized by abnormalities in PDGFRA, IDH1, EGFR, and NF1. Cancer Cell.

[3] Noushmehr H., Weisenberger D.J., Diefes K., Phillips H.S., Pujara K., Berman B.P., Pan F., Pelloski C.E., Sulman E.P., Bhat K.P. (2010). Identification of a CpG island methylator phenotype that defines a distinct subgroup of glioma. Cancer Cell.

[4] Turcan S., Rohle D., Goenka A., Walsh L.A., Fang F., Yilmaz E., Campos C., Fabius A.W.M., Lu C., Ward P. (2012). IDH1 mutation is sufficient to establish the glioma hypermethylator phenotype. Nature.

[5] Kraboth Z., Galik B., Tompa M., Kajtar B., Urban P., Gyenesei A., Miseta A., Kalman B. (2020). DNA CpG methylation in sequential glioblastoma specimens. J. Cancer. Res. Clin. Oncol..

[6] Kraboth Z., Kajtár B., Gálik B., Gyenesei A., Miseta A., Kalman B. (2021). Involvement of the Catecholamine Pathway in Glioblastoma Development. Cells.

[7] Tompa M., Kajtar B., Galik B., Gyenesei A., Kalman B. (2021). DNA methylation and protein expression of Wnt pathway markers in progressive glioblastoma. Pathol. Res. Pract..

[8] Najem H., Khasraw M., Heimberger A.B. (2021). Immune Microenvironment Landscape in CNS Tumors and Role in Responses to Immunotherapy. Cells.

[9] Liu X., Xing H., Liu H., Chen J. (2022). Current status and future perspectives on immunotherapy in neoadjuvant therapy of resectable non-small cell lung cancer. Asia. Pac. J. Clin. Oncol..

[10] Ossato A., Damuzzo V., Baldo P., Mengato D., Chiumente M., Messori A. (2022). Immune checkpoint inhibitors as first line in advanced melanoma: Evaluating progression-free survival based on reconstructed individual patient data. Cancer Med..

[11] Sanders S., Debinski W. (2020). Challenges to successful implementation of the immune checkpoint inhibitors for treatment of glioblastoma. Int. J. Mol. Sci..

[12] Louis D.N., Perry A., Reifenberger G., Von Deimling A., Figarella-Branger D., Cavenee W.K., Ohgaki H., Wiestler O.D., Kleihues P., Ellison D.W. (2016). The 2016 World Health Organization classification of tumors of the central nervous system: A summary. Acta. Neuropathol..

[13] Louis D.N., Perry A., Wesseling P., Brat D.J., Cree I.A., Figarella-Branger D., Hawkins C., Ng H.K., Pfister S.M., Reifenberger G. (2021). The 2021 WHO Classification of Tumors of the Central Nervous System: A summary. Neuro-Oncology.

[14] Europian Nucleotide Archive; Primary Accession: PRJNA391429; EGAS00001002538. https://www.ebi.ac.uk/ena.

[15] National Cancer Institute, GDC Data Portal, TCGA-GBM database. https://portal.gdc.cancer.gov/repository?filters=%7B%22op%22%3A%22and%22%2C%22content%22%3A%5B%7B%22op%22%3A%22in%22%2C%22content%22%3A%7B%22field%22%3A%22cases.project.project_id%22%2C%22value%22%3A%5B%22TCGA-GBM%22%5D%7D%7D%2C%7B%22op%22%3A%22in%22%2C%22content%22%3A%7B%22field%22%3A%22files.data_category%22%2C%22value%22%3A%5B%22DNA%20Methylation%22%5D%7D%7D%5D%7D&searchTableTab=cases.

[16] Klughammer J., Kiesel B., Roetzer T., Fortelny N., Nemc A., Nenning K.H., Furtner J., Sheffield N.C., Datlinger P., Peter N. (2018). The DNA methylation landscape of glioblastoma disease progression shows extensive heterogeneity in time and space. Nat. Med..

[17] Pearson J.R., Cuzzubbo S., McArthur S., Durrant L.G., Adhikaree J., Tinsley C.J., Pockley A.G., McArdle S.E.B. (2020). Immune escape in glioblastoma multiforme and the adaptation of immunotherapies for treatment. Front. Immunol..

[18] Mescher M.F., Curtsinger J.M., Agarwal P., Casey K.A., Gerner M., Hammerbeck C.D., Popescu F., Xiao Z. (2006). Signals required for programming effector and memory development by CD8+ T cells. Immunol. Rev..

[19] Shum T., Omer B., Tashiro H., Kruse R.L., Wagner D.L., Parikh K., Yi Z., Sauer T., Liu D., Parihar R. (2017). Constitutive signaling from an engineered IL7 receptor promotes durable tumor elimination by tumor-redirected T cells. Cancer Discov..

[20] Gangoso E., Southgate B., Bradley L., Rus S., Galvez-Cancino F., McGivern N., Güç E., Kapourani C.-A., Byron A., Ferguson K.M. (2021). Glioblastomas acquire myeloid-affiliated transcriptional programs via epigenetic immunoediting to elicit immune evasion. Cell.

[21] Sawada M., Itoh Y., Suzumura A., Marunouchi T. (1993). Expression of cytokine receptors in cultured neuronal and glial cells. Neurosci. Lett..

[22] Mazzucchelli R., Durum S.K. (2007). Interleukin-7 receptor expression: Intelligent design. Nat. Rev. Immunol..

[23] Huang J., Zheng M., Zhang Z., Tang X., Chen Y., Peng A., Peng X., Tong A., Zhou L. (2021). Interleukin-7-loaded oncolytic adenovirus improves CAR-T cell therapy for glioblastoma. Cancer. Immunol. Immunother..

[24] Noguchi M., Nakamura Y., Russell S.M., Ziegler S.F., Tsang M., Cao X., Leonard W.J. (1993). Interleukin-2 receptor gamma chain: A functional component of the interleukin-7 receptor. Science.

[25] Perna S.K., Pagliara D., Mahendravada A., Liu H., Brenner M.K., Savoldo B., Dotti G. (2014). Interleukin-7 mediates selective expansion of tumor-redirected cytotoxic T lymphocytes (CTLs) without enhancement of regulatory T-cell inhibition. Clin. Cancer Res..

[26] Baizan-Edge A., Stubbs B.A., Stubbington M.J.T., Bolland D.J., Tabbada K., Andrews S., Corcoran A.E. (2021). IL-7R signaling activates widespread VH and DH gene usage to drive antibody diversity in bone marrow B cells. Cell Rep..

[27] Lin K., Gueble S.E., Sundaram R.K., Huseman E.D., Bindra R.S., Herzon S.B. (2022). Mechanism-based design of agents that selectively target drug-resistant glioma. Science.

[28] Cui G., Shimba A., Ma G., Takahara K., Tani-Ichi S., Zhu Y., Asahi T., Abe A., Miyachi H., Kitano S. (2020). IL-7R-Dependent Phosphatidylinositol 3-Kinase Competes with the STAT5 Signal to Modulate T Cell Development and Homeostasis. J. Immunol..

[29] Grossman S.A., Ye X., Lesser G., Sloan A., Carraway H., Desideri S., Piantadosi S., NABTT CNS Consortium (2011). Immunosuppression in patients with high-grade gliomas treated with radiation and temozolomide. Clin. Cancer Res..

[30] Rosenberg S.A., Sportès C., Ahmadzadeh M., Fry T.J., Ngo L.T., Schwarz S.L., Stetler-Stevenson M., Morton K.E., Mavroukakis S.A., Morre M. (2006). IL-7 administration to humans leads to expansion of CD8+ and CD4+ cells but a relative decrease of CD4+ T-regulatory cells. J. Immunother..

[31] Ma X., Shou P., Smith C., Chen Y., Du H., Sun C., Kren N.P., Michaud D., Ahn S., Vincent B. (2020). Interleukin-23 engineering improves CAR T cell function in solid tumors. Nat. Biotechnol..

[32] Khan M.D.A., Zheng M., Fu J., Tania M., Li J., Fu J. (2022). Thymoquinone upregulates IL17RD in controlling the growth and metastasis of triple negative breast cancer cells in vitro. BMC Cancer.

[33] Mondal P., Natesh J., Penta D., Meeran S.M. (2022). Progress and promises of epigenetic drugs and epigenetic diets in cancer prevention and therapy: A clinical update. Semin. Cancer Biol..

[34] Zheng Z., Huang G., Gao T., Huang T., Zou M., Zou Y., Duan S. (2020). Epigenetic Changes Associated with Interleukin-10. Front. Immunol..

[35] Rodrigues G.O.L., Cramer S.D., Winer H.Y., Hixon J.A., Li W., Yunes J.A., Durum S.K. (2021). Mutations that collaborate with IL-7Ra signaling pathways to drive ALL. Adv. Biol. Regul..

[36] Ronvaux L., Riva M., Coosemans A., Herzog M., Rommelaere G., Donis N., D’Hondt L., Douxfils J. (2022). Liquid Biopsy in Glioblastoma. Cancers.

